# Validity and reliability of the 2-min step test in individuals with stroke and lower-limb musculoskeletal disorders

**DOI:** 10.3389/fresc.2024.1384369

**Published:** 2024-04-16

**Authors:** Tomoya Ishigaki, Hiroki Kubo, Keishi Yoshida, Natsuki Shimizu, Tatsuya Ogawa

**Affiliations:** ^1^ Department of Physical Therapy, Faculty of Rehabilitation Sciences, Nagoya Gakuin University, Aichi, Japan; ^2^ Department of Physical Therapy, Faculty of Nursing and Rehabilitation, Konan Women’s University, Hyogo, Japan; ^3^ Department of Rehabilitation, Senri-Chuo Hospital, Osaka, Japan; ^4^ Department of Physical Therapy, Faculty of Health and Medical Care, Saitama Medical University, Saitama, Japan; ^5^ Department of Rehabilitation, Nishiyamato Rehabilitation Hospital, Nara, Japan

**Keywords:** stroke, lower-limb musculoskeletal disorder, exercise capacity, exercise endurance, two-minute step test

## Abstract

**Introduction:**

We investigated the reliability and validity of the 2-min step test (2MST) for assessing the exercise endurance of individuals with stroke and lower-limb musculoskeletal disorders.

**Participants and methods:**

The participants were 39 individuals with stroke and 42 with lower-limb musculoskeletal disorders (mainly hip fractures) from the convalescent rehabilitation wards of four hospitals. The concurrent validity and congruence between the 2MST and the 6-min walk test (6MWT) and construct validity by hypotheses testing, including mobility and lower limb muscle strength, were also confirmed. A subset of participants (stroke-group, *n* = 15; musculoskeletal-group, *n* = 19) underwent a retest 2MST for our evaluation of relative and absolute reliability using the intraclass correlation coefficient (ICC_1,1_) and Bland–Altman plot.

**Results:**

Both groups showed a moderate correlation between the 2MST and 6MWT (*ρ* = 0.55–0.60), but the congruence was not sufficient. The 6MWT was correlated with mobility in both groups and with muscle strength in the stroke group, whereas the 2MST did not show a significant correlation with mobility. The relative reliability was excellent in both groups (ICC_1,1_ > 0.9). In terms of absolute reliability, the width of the limit of agreement was 18.8% for the stroke group and 15.4% for the musculoskeletal group, relative to their respective sample means of 2MST. A fixed bias was identified in the stroke group, in which step counts increased by 6.5 steps upon retesting.

**Discussion:**

Our analyses revealed that the 2MST is a valid and reliable tool for assessing the exercise endurance of individuals with stroke or lower-limb musculoskeletal disorders. However, it is necessary to validate the absolute reliability observed herein by using a larger sample size. In addition, when assessing the exercise endurance of individuals with stroke, it may be necessary to consider the potential bias of an increased step count during retesting.

## 1 Introduction

Exercise capacity is a defining factor of physical fitness and a crucial determinant of successful aging (1). Numerous studies have shown a dose-response relationship between increased exercise capacity and reduced morbidity and mortality in older adults (2). Consequently, the World Health Organization's physical activity guidelines recommend aerobic exercise for a variety of individuals, including adults, older adults, and those with chronic disease and disability (3). It is therefore essential to evaluate individuals' exercise capacity properly to determine the advantages of aerobic exercise. Exercise capacity is typically assessed by using the testee's maximal oxygen uptake, which can be measured using direct or indirect methods. The direct method involves analyzing exhaled gas during exercise on a treadmill or bicycle ergometer, which requires specialized equipment, space, and trained professionals. In contrast, the indirect method estimates maximal oxygen uptake based on the amount of exercise (i.e., exercise endurance) that can be performed within a time limit. Although direct methods can accurately assess exercise capacity, indirect methods based on exercise endurance are often used in clinical settings due to their broad and simple applicability. The most common indirect method is the 6-min walk test (6MWT), which measures the distance a subject can walk in a 6-min period (4). The 6MWT is a standard clinical assessment recommended in practice guidelines or evidence reviews for evaluating exercise endurance. This test is applicable to conditions such as stroke (5) and lower-limb musculoskeletal disorders (LMSD), including hip fractures (6), knee or hip osteoarthritis, and total knee or hip arthroplasty (7), which may affect the activities of daily living of older adults. However, due to the requirement of a long walkway, it may not be feasible to perform the 6MWT in clinics, homes, or other clinical settings with limited space. In Japan, where the population is the most aged among major industrialized countries (8), stroke and LMSD (including fractures, falls, and joint diseases) are the major causes of the need for long-term care (9). Against the backdrop of the aging population in Japan, the government is promoting a shift in medical and nursing care (including rehabilitation services) from hospitals to homes as a matter of policy (10). In other words, there is a need to establish a simple method to assess the exercise endurance of individuals with stroke or LMSD in home and community settings, which are more environmentally constrained than in hospitals. Moreover, aging is a global concern that is not unique to Japan (11), and evidence for telerehabilitation performed in the home setting has been building as a matter of global concern (12, 13). Therefore, addressing this issue holds significance not only for Japan but worldwide.

An alternative to the 6MWT, which requires less space, is the 2-min step test (2MST) (14). The 2MST was developed as a subtest of the Senior Fitness Test and is a method for assessing exercise endurance (14–16). In the 2MST, the subject assumes a standing position and performs as many marching movements as possible for 2 min on the spot. Performance on the 2MST is defined by measuring the number of unilateral (usually right-sided) steps taken in the standing position to a height midway between the patella and iliac crest, with a higher number indicating greater exercise endurance. The 2MST was originally designed for older adults, but recent studies have shown its validity as an exercise endurance assessment tool in various populations, including older adults (14, 17) and those with cardiovascular diseases (18–20), Parkinson's disease (21), symptomatic peripheral artery disease (22), type 2 diabetes (23), hypertension (24), and morbid obesity (25). The inter-and intra-rater reliabilities of the 2MST have been reported in various populations, including older adults (14, 16), young to middle-aged adults (26), and individuals with cardiovascular diseases (20), symptomatic peripheral arterial diseases (22), chronic low back pain (27), and knee osteoarthritis (28).

However, the reliability and validity of the 2MST in individuals with stroke and LMSD, including hip fracture and knee or hip arthroplasty, have not been adequately investigated. We conducted the present study to investigate the reliability and validity of the 2MST as an assessment of the exercise endurance of individuals with stroke and LMSD.

## 2 Participants and methods

### 2.1 Study design, ethics and reporting guideline

This study was a multicenter, cross-sectional survey. The study was approved by the Medical Ethics Committee of Nagoya Gakuin University (approval no. 2020-28). The study complied with the Declaration of Helsinki, and all participants provided written informed consent. This study evaluated the measurement properties of the 2MST according to the taxonomy developed by the Consensus-based Standards for the selection of health Measurement INstruments (COSMIN) initiative and reported them in accordance with the reporting guidelines developed by COSMIN (29, 30).

### 2.2 Study setting and participants

This study was conducted in the convalescent rehabilitation wards of four hospitals in Japan from August 2021 to August 2022, where volunteers were recruited to participate. The study targeted individuals with first-time stroke (infarction or hemorrhage) or an LMSD (hip or femoral fracture, hip or knee osteoarthritis, or total knee arthroplasty). The inclusion criteria were: (*i*) age ≥45 years, (*ii*) ≥60 days post-stroke onset and ≥45 days post-onset of injury or hospitalization due to an LMSD, (*iii*) overall stable health condition with no exercise restrictions imposed by the attending physician related to the expected exercise load in this study, and (*iv*) ability to walk with supervision using a walking aid or lower-limb orthosis. The exclusion criteria were: (*i*) comorbidity requiring the management of cardiac or respiratory illnesses; (*ii*) the presence of acute pain; and (*iii*) cognitive impairments, consciousness disorders, or mental illnesses that would hinder participation in the study.

To calculate the sample size, we determined the concurrent validity based on the correlation coefficient between the 2MST and 6MWT. In reference to a study of individuals with heart failure reporting a correlation coefficient of 0.44 between the 2MST and the 6MWT (18), a sample size of 38 participants for each of the present groups (stroke and LMSD) was calculated, considering a significance level of 0.05 and a power of 0.80. During the planning phase of the research proposal, this study (18) was the only one that validated the correlations between 2MST and 6MWT and peak oxygen uptake among middle-aged and older individuals with diseases. Therefore, although the disease differs from that in our study, it was used as a reference value to calculate the sample size. The minimum sample size was 46 participants per group, with a 20% anticipated data loss. This calculation was performed using G*Power 3.1.9.6 [test family: exact; statistical test: correlation (bivariate normal model)] (31). Reliability data were randomly selected from the participants who provided data for validity. Reliability was determined based on an intra-rater reliability coefficient [intraclass correlation coefficient (ICC_1,1_)] of 0.7, using the test-retest method, with a significance level of 0.05, and power of 0.80. This resulted in a required sample size of 12 participants for each group. Assuming a 30% data loss, a minimum sample size of 16 participants was planned for each group. The R package (ICC.Sample.Size) was used for this calculation (32). Participant recruitment was stopped early when sufficient valid data were obtained for the calculated minimum sample size.

### 2.3 Data collection

The 2MST and 6MWT were conducted on different days for each participant, ranging from ≥1 day to <7 days apart. The examiner was given discretion to choose which test to perform first. Only randomly selected participants, chosen for the examination of reliability, underwent the 2MST again within a 7-day period. The researchers, who are licensed physiotherapist, agreed that neither of the day intervals would result in changes in the participants' conditions that could influence the test results. Data were collected by physiotherapists who were informed of the purpose, content, and methods of the study. There were no restrictions on data collection by the physiotherapists who handled the patients during their regular clinical duties. Demographic and clinical characteristics were collected on the day the 2MST or 6MWT was conducted for the first time. The participants were not blinded to their 2MST or 6MWT results.

#### 2.3.1 Demographics and clinical characteristics

Data on age, sex, body mass index (BMI), days from onset, type of stroke or LMSD, affected side, and site(s) were collected from the participants' medical records. Comorbidities contributing to mortality were evaluated and scored using the Charlson Comorbidity Index (33); the CCI scoring used an updated version of the index rather than the original version (34). The scores range from 0 to 24 points, with higher scores indicating a greater impact of comorbid conditions and an increased risk of mortality.

#### 2.3.2 Ambulation ability and mobility

We also evaluated the ambulation ability and mobility. Ambulation ability was assessed using the Functional Ambulation Categories (FAC) scale, which ranges from non-functional ambulator to independent ambulator, with six stages (0–5) (35). A higher FAC stage indicates greater ambulation ability. The Japanese version of the Rivermead Mobility Index (RMI) was used to assess mobility. The RMI evaluates independence in 15 aspects of mobility, including bed mobility, transfers, walking, bathing, and stairs, and is scored from 0 (poorest) to 15 (best) (36, 37).

#### 2.3.3 Physical functions

Pain intensity during walking was evaluated using a Numeric Rating Scale (NRS) ranging from 0 (no pain) to 10 (maximum pain) (38). Lower-limb muscle strength, specifically hip flexion and knee extension, was assessed using manual muscle testing on both sides in six stages (39). However, for the affected side of the stroke participants and the muscle strength of the affected side (including hip flexion, knee extension, and ankle dorsiflexion) was assessed using the Motricity Index (40), which is comprised of six stages for each muscle; the total scores were calculated on a scale ranging from 1 (poorest) to 100 (best) (41).

#### 2.3.4 Exercise endurance

The 6MWT was conducted in accord with the guidelines of the American Thoracic Society, using a 30-meter walkway (42). The participant was instructed to walk as far as possible within 6 min, with breaks allowed as needed during the test. When taking a break, the participants were encouraged to resume walking as quickly as possible. The total distance walked was recorded.

The 2MST was conducted in accord with the procedures of the Senior Fitness Test (14), and the participant was instructed to march as many steps as possible for 2 min on the spot. To set the elevation height of the lower limbs, the midpoint between the patella and the anterior superior iliac spine of each participant was identified. If a participant had difficulty raising the affected limb or the more severely affected side to a standard height, he or she was instructed to raise it to the best of their ability. The number of steps taken over a period of 2 min was then measured based on the non-affected or less affected limb. We excluded individuals who were unable to elevate to the set height due to physical limitations. To ensure the safety of participants with balance disorders and to maintain uniform testing conditions, all tests were conducted with the participants holding onto a handrail with one hand. The original manual for 2MST also mentions the option to allow the use of handrail (14).

For both the 6MWT and 2MST, the % Heart Rate Reserve (%HRR) was calculated by measuring the participant's heart rate before and after completing the exercise tasks. The modified Borg scale (0–10) was used to assess the rate of perceived exertion (RPE) following the exercise tasks (43).

### 2.4 Statistical analyses

Statistical analyses were conducted separately for the stroke and LMSD groups. To understand the characteristics of the sample, we calculated descriptive statistics based on the scale properties of each variable and data distribution. Normality was examined using histograms, Q–Q plots, and the Shapiro–Wilk test. Means and standard deviations were used to describe interval scale variables that were confirmed to be normal, whereas medians and first and third quartiles were used for those that were not confirmed to be normal. Nominal scale variables are presented as frequencies and percentages.

We investigated the validity and reliability of the 2MST as a measure of exercise endurance. For validity, both the concurrent validity and agreement between the 2MST and the 6MWT as well as construct validity by hypotheses testing were evaluated. Reliability was assessed by an examination of the intra-rater reliability using the test-retest method, focusing on both relative and absolute reliability. The statistical analyses were performed with R 4.3.1 (CRAN) using the Shrout method for the ICC (44) and the Stratford method for the standard error of measurement (SEM) (45), with a significance level of 5%.

#### 2.4.1 Validity

We used Spearman's rank correlation coefficient to examine the concurrent validity of the 2MST and 6MWT. A non-parametric method was employed for this purpose to align with the methodology used in the subsequent analyses of construct validity. In addition, to quantitatively assess the congruence between the 2MST and 6MWT, a simple regression analysis was performed to predict the 6MWT results from the 2MST results, and a 95% prediction interval at the mean value of the 2MST was determined (46).

To verify and compare the construct validity of the 2MST and 6MST, we examined their relationships with mobility (RMI), pain intensity (NRS), and the strength of the affected lower limb (MMT and Motricity Index) using Spearman's rank correlation coefficient for each group. The construct validity hypothesis was as follows: 2MST, an on-the-spot marching exercise performed while holding a handrail and counting movements of the unaffected side, was hypothesized to have little or no correlation with mobility, pain intensity, or the strength of the affected lower limb. In contrast, the 6MWT, which involves walking, was expected to have a higher correlation with mobility, the strength of the affected lower limb, and pain during walking. In other words, the 2MST was hypothesized to be less influenced by walking or walking-related physical functions in its assessment of exercise endurance. Because we conducted multiple correlation analyses for both concurrent and construct validity, the probability (*p*)-values were adjusted using the Holm method to account for the risk of alpha error. The interpretation of the correlation coefficient was defined as follows: 0.0 to ±0.1 as negligible, ±0.1 to ±0.39 as weak, ±0.4 to ±0.69 as moderate, ±0.7 to ±0.89 as strong, and ±0.9 to ±1.0 as very strong (47).

#### 2.4.2 Reliability

To evaluate the relative reliability of the 2MST, we used the ICC_1,1_ to analyze the correlation coefficient between the initial test and retest, and the SEM was also determined. The interpretation of ICC was defined as follows: <0.5 as poor, 0.5–0.75 as moderate, 0.75–0.9 as good, and >0.90 as excellent reliability (48).

As a secondary outcome of reliability, we examined absolute reliability, with the aim of providing reference values for future research. The systematic error between the initial test and retest in the 2MST was assessed using Bland–Altman plots (49). Following the reporting framework (50) recommended in a recent review (51), our analysis confirmed the normality of the mean and the difference between two values (initial and retest) using Q–Q plots and the Shapiro–Wilk test. Subsequently, we calculated the mean of the differences with their 95% confidence intervals (CIs), as well as the limits of agreement (LoA) and their upper and lower 95% CIs. Fixed bias was examined using the mean of the difference, 95% CIs, and a one-sample *t*-test, whereas proportional bias was assessed based on the significance of the Pearson's product-moment correlation coefficient. These analyses based on Bland–Altman plots were performed using the web tool provided by Olofsen et al. (52), with a detailed methodology described in their paper (53). The LoA for the 2MST performed by individuals with stroke or LMSD has not yet been reported; therefore, as an alternative, in the present study we assumed that the LoA for the 6MWT of the participants with stroke or hip fracture (ranges corresponding to ±35% and ±18% of the sample mean, respectively) were within acceptable ranges (54, 55).

## 3 Results

### 3.1 Characteristics of the participants

In the stroke group, 43 individuals who met the criteria participated in the study, but four were excluded due to an improper administration of either the 6MWT or 2MST. The final sample consisted of 39 individuals for the validity analysis and 15 for the reliability analysis. In the LMSD group, 42 individuals who met the criteria participated. Individuals who had undergone a total hip arthroplasty did not participate in this study. One participant was excluded due to an improper administration of 2MST. The final sample consisted of 42 individuals for validity and 19 individuals for reliability.

The descriptive statistics for each dataset in both groups are presented in Tables 1, 2. The stroke group consisted of middle-aged to older adults, with a slightly higher number of males suffering from cerebral infarction. At least 70% of the participants in the stroke group were able to walk independently within the hospital (FAC ≥ 4). The LMSD group mostly included older females with hip fractures who were almost (≥95%) independently ambulatory within the hospital (FAC ≥ 4). In both the stroke and LMSD groups, for the validity and reliability datasets, the walking distance in the 6MWT was approx. 320 m. In contrast, in the 2MST, the average number of steps for the stroke group in the validity data was 78, compared with 91 in the LMSD group, which was slightly higher. However, in the reliability dataset, both groups exhibited an average of 94–100 steps.

**Table 1:** Characteristics of the participants in the stroke group.

Variable	Validity data *n* = 39	Reliability data *n* = 15
Age, yrs	65.0 (56.0–78.0)	58.0 (48.0–74.0)
Sex: Male/female, *n* (%)	25 (64.1)/14 (35.9)	9 (60.0)/6 (40.0)
BMI, kg/m^2^	22.9 (20.3–24.7)	22.0 (20.5–23.4)
Type of stroke: Infarction/hemorrhage, *n* (%)	24 (61.5)/15 (38.5)	10 (66.7)/5 (33.3)
Affected side: Right/left/bilateral, *n* (%)	19 (48.7)/19 (48.7)/1 (2.6)	6 (40.0)/9 (60.0)/0 (0)
CCI update, points	2 (2–2)	2 (2–2)
FAC, grade 3/4/5, *n* (%)	10 (25.6)/17 (43.6)/12 (30.8)	4 (26.7)/7 (46.7)/4 (26.7)
RMI, points	9 (8–11)	9 (8–12)
NRS pain walking, points	0 (0–0)	0 (0–0)
Motricity index, points:	76 (58–92)	65 (43–84)
Motricity hip, points 9/14/19/25/33	1 (2.6)/6 (15.4)/9 (23.1)/16 (41.0)/7 (17.9)	1 (6.7)/3 (20.0)/4 (26.7)/5 (33.3)/2 (13.3)
Motricity knee, points 9/14/19/25/33	2 (5.1)/4 (10.3)/5 (12.8)/17 (43.6)/11 (28.2)	2 (13.3)/3 (20.0)/2 (13.3)/4 (26.7)/4 (26.7)
Motricity ankle, points 0/9/14/19/25/33	1 (2.6)/3 (7.7)/ 6 (15.4)/5 (12.8)/12 (30.8)/12 (30.8)	1 (6.7)/2 (13.3)/ 3 (20.0)/2 (13.3)/3 (20.0)/4 (26.7)
Non-as hip.flex MMT, grade 4/5, *n* (%)	14 (35.9)/25 (64.1)	4 (26.7)/11 (73.3)
Non-as knee.ext MMT, grade 4/5, *n* (%)	9 (23.1)/30 (76.9)	0 (0)/15 (100)
6MWT, m:	314.0 ± 111.8	324.4 ± 123.6
6MWT RPE, points	4 (2–4)	4 (2–4)
6MWT %HRR (%)	18.6 (10.1–29.1)	15.8 (7.8–27.9)
Days from onset to 6MWT	106.4 ± 37.3	114.7 ± 35.3
2MST, steps:	78.4 ± 25.8	94.4 ± 26.3
2MST RPE, points	4 (3–5)	4 (3–5)
2MST %HRR (%)	19.8 (13.5–27.9)	21.2 (13.5–27.0)
Days from onset to 2MST	105.5 ± 36.5	111.7 ± 33.6
Days from 6MWT to 2MST	2 (1–4)	3 (1–5)
Retest 2MST, steps:	–	100.9 ± 30.7
Retest 2MST RPE, points	–	4 (2–5)
Retest 2MST %HRR (%)	–	19.1 (11.8–29.1)
Days from test to retest 2MST	–	7 (7–7)

Mean ± standard deviation, median (1st–3rd quartile).

%HRR, % heart rate reserve; 2MST, 2-min step test; 6MWT, 6-min walk test; BMI, body mass index; CCI, Charlson comorbidity index; FAC, functional ambulation categories; MMT, manual muscle testing; Non-As, non-affected side; NRS pain walking, numerical rating scale of pain intensity in walking; RMI, rivermead mobility index; RPE, rating of perceived exertion.

**Table 2:** Characteristics of the participants in the lower-limb musculoskeletal disorders (LMSD) group.

Variable	Validity data *n* = 42	Reliability data *n* = 19
Age, years	79.0 (71.0–83.0)	78.0 (75.0–82.0)
Sex: Male/female, *n* (%)	7 (16.7)/35 (83.3)	4 (21.1)/15 (78.9)
BMI, kg/m^2^	20.8 (19.0–24.3)	20.8 (19.1–23.2)
Type of LMSD: Fractures hip/femoral Osteoarthritis hip/knee Total knee arthroplasty, *n* (%)	31 (73.8)/2 (4.8) 2 (4.8)/5 (11.9) 2 (4.8)	14 (73.7)/1 (5.3) 1 (5.3)/3 (15.8) 0 (0)
Affected side: Right/left/bilateral, *n* (%)	16 (38.1)/25 (59.5)/1 (2.4)	5 (26.3)/13 (68.4)/1 (5.3)
Affected of site: Hip/knee, *n* (%)	35 (83.3)/7 (16.7)	16 (84.2)/3 (15.8)
CCI update, points	0 (0–1)	0 (0–0)
FAC, grade 3/4/5, *n* (%)	2 (4.8)/24 (57.1)/16 (38.1)	0 (0)/14 (73.7)/5 (26.3)
RMI, points	9 (8–9)	9 (8–9)
NRS pain walking, points	1 (0–2)	2 (1–3)
As hip.flex MMT, grade 3/4/5, *n* (%)	10 (23.8)/26 (61.9)/6 (14.3)	5 (26.3)/12 (63.2)/2 (10.5)
As knee.ext MMT, grade 2/3/4/5, *n* (%)	1 (2.4)/7 (16.7)/21 (50.0)/13 (31.0)	0 (0)/5 (26.3)/9 (47.4)/5 (26.3)
Non-AS hip.flex MMT, grade 3/4/5, *n* (%)	2 (4.8)/27 (64.3)/13 (31.0)	1 (5.3)/12 (63.2)/6 (31.6)
Non-AS knee.ext MMT, grade 3/4/5, *n* (%)	3 (7.1)/21 (50.0)/18 (42.9)	1 (5.3)/10 (52.6)/8 (42.1)
6MWT, m:	320.0 ± 87.5	327.6 ± 72.7
6MWT RPE, points	4 (3–5)	4 (3–5)
6MWT %HRR (%)	16.4 (10.2–23.9)	14.9 (9.7–18.5)
Days from onset to 6MWT	67.2 ± 20.9	66.4 ± 14.6
2MST, steps:	91.1 ± 24.5	95.7 ± 29.5
2MST RPE, points	4 (3–6)	3 (2–4)
2MST %HRR (%)	19.0 (12.6–32.8)	14.6 (7.3–20.4)
Days from onset to 2MST	65.2 ± 20.7	63.9 ± 14.4
Days from 6MWT to 2MST	2 (1–3.3)	3 (1–8)
Retest 2MST, steps:	–	97.2 ± 28.7
Retest 2MST RPE, points	–	4 (3–4)
Retest 2MST %HRR (%)	–	10.5 (6.5–18.6)
Days from test to retest 2MST	–	7 (7–8)

Mean ± standard deviation, median (1st–3rd quartile).

%HRR, % heart rate reserve; 2MST, 2-min step test; 6MWT, 6-min walk test; BMI, body mass index; CCI, Charlson comorbidity index; FAC, functional ambulation categories; MMT, manual muscle testing; Non-As, non-affected side; NRS pain walking, numerical rating scale of pain intensity in walking; RMI, rivermead mobility index; RPE, rating of perceived exertion.

### 3.2 Validity and congruence

**Figure 1:**
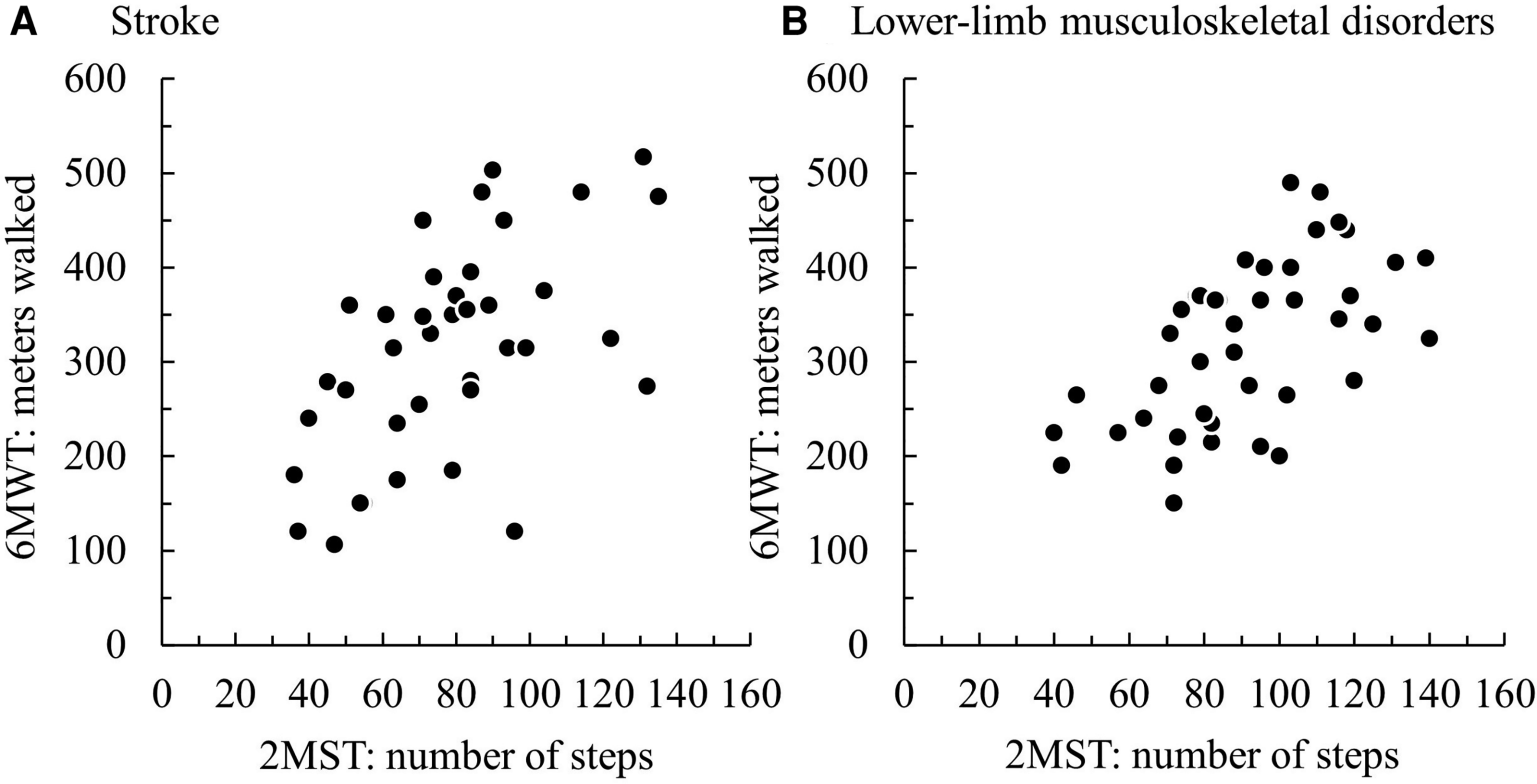
Scatterplots of the participants' results on the 2-min step test (2MST) and 6-min walk test (6MWT) in (**A**) the group with stroke and (**B**) the group with lower-limb musculoskeletal disorders.

**Figure 2:**
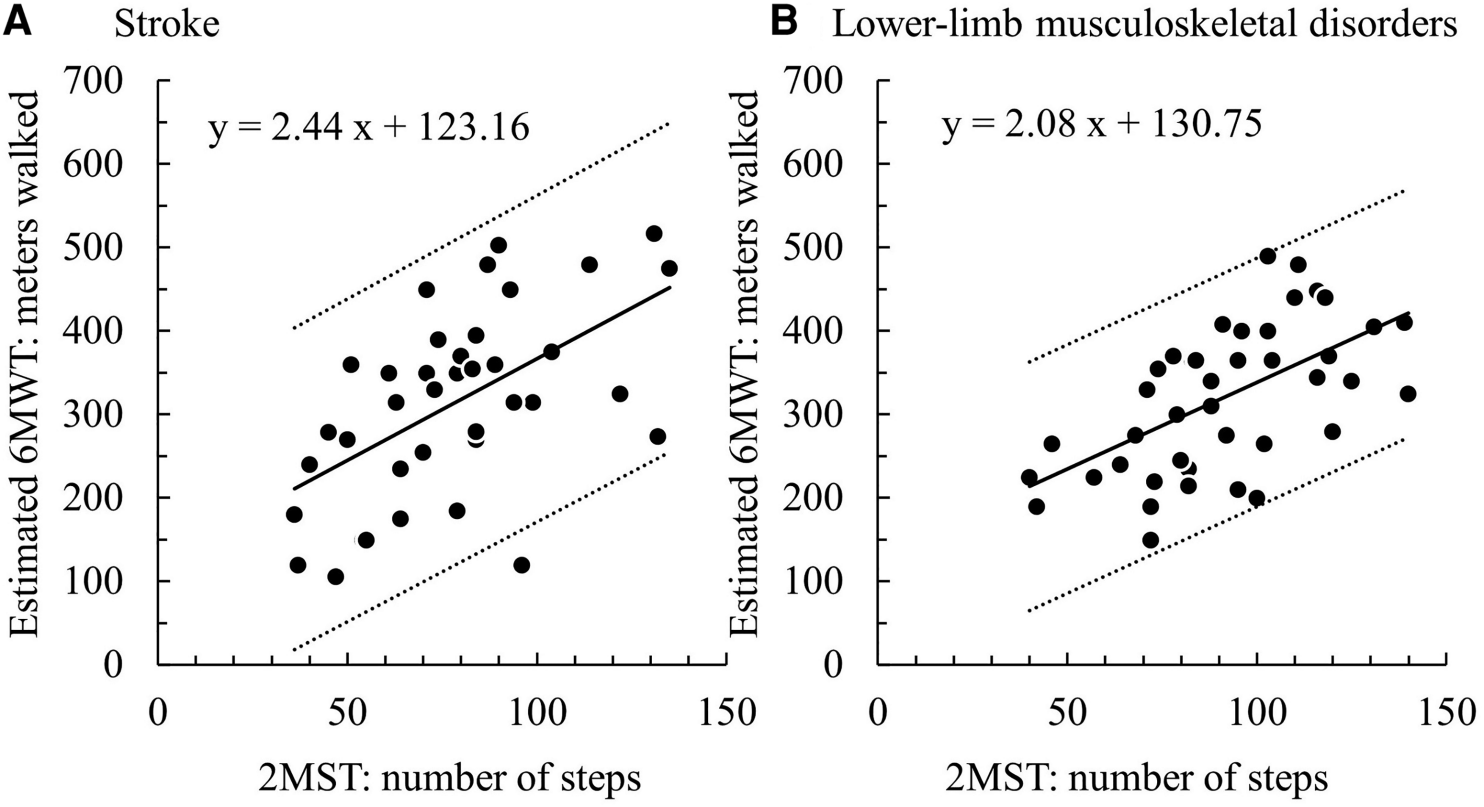
(**A**) stroke group: 95% prediction interval = 371.8 m (128.1–499.9), (**B**) lower-limb musculoskeletal disorders group: 95% prediction interval = 285.7 m (177.1–462.9).

Regarding concurrent validity, significant moderate correlations between the 2MST and the 6MWT were observed in both groups (stroke *ρ* = 0.55, *p* < 0.01; LMSD *ρ* = 0.60, *p* < 0.01) (Figures 1A,B and Table 3). Table 4 presents the results of the simple regression analysis estimating 6MWT from 2MST for each group. For the congruence between the 2MST and 6MWT, in the stroke group, the 95% prediction interval for the mean value of the 2MST was between a lower bound of 177.1 m and an upper bound of 462.9 m, with a range of 285.7 m (Figure 2A). In the LMSD group, the 95% prediction interval for the mean value of the 2MST was between a lower limit of 128.1 m and an upper limit of 499.9 m, with a range of 371.8 m (Figure 2B). The range of the predictive interval was wide in both groups, ranging from ±45% to 58% of mean 6MWT. Regarding construct validity, the 6MWT showed a significant moderate correlation with the RMI (mobility) result in both groups (stroke *ρ* = 0.51, *p* < 0.01; LMSD, *ρ* = 0.43, *p* < 0.01). In the stroke group, a significant moderate correlation was observed between the Motricity Index (the strength of the affected lower limb) (*ρ* = 0.67, *p* < 0.01). However, the 2MST did not show any significant correlation with these variables (Table 3). It should be noted that because of the very low incidence of pain in the stroke group, pain was not included in the analysis for this group.

**Table 3:** The 2MST and the 6MWT and correlations between each variable.

	Stroke (*n* = 39)		LMSD (*n* = 42)	
	2MST	6MWT	2MST	6MWT
6MWT	0.55 (< 0.01)	–	0.60 (<0.01)	–
RMI	0.18 (0.17)	0.51 (<0.01)	0.14 (1.00)	0.43 (0.04)
Motricity index	0.28 (0.25)	0.67 (<0.01)	–	–
NRS pain walking	–	–	−0.17 (1.00)	−0.13 (0.83)
As hip.flex MMT	–	–	0.33 (0.21)	0.33 (0.23)
As knee.ext MMT	–	–	−0.03 (0.85)	0.20 (0.99)

Spearman's rank correlation coefficient (*p*-value).

2MST, 2-min step test; 6MWT, 6-min walk test; As, affected side; LMSD, lower-limb musculoskeletal disorders; MMT, manual muscle testing; NRS pain walking, numerical rating scale of pain intensity in walking; RMI, rivermead mobility index.

**Table 4:** Results of single regression analysis to estimate 6MWT from 2MST.

Variable	Stroke *n* = 15			LMSD *n* = 19		
	Coefficient	SE (95% CI)	*p*-value	Coefficient	SE (95% CI)	*p*-value
Intercept	123.16	48.49 (24.90–221.41)	<0.001	130.75	43.32 (43.18–218.31)	0.02
2MST	2.44	0.59 (1.24–3.63)	<0.001	2.08	0.46 (1.15–3.01)	<0.001

Stroke: Adjusted *R*^2^ = 0.30, ANOVA *p* < 0.001, Variance of residuals = 8,771.19; Musculoskeletal: Adjusted *R*^2^ = 0.32, ANOVA *p* < 0.001, Variance of residuals = 5,190.13.

6MWT, 6-min walk tes; 2MST, 2-min step test; LMSD, lower-limb musculoskeletal disorders.

### 3.3 Reliability

**Figure 3:**
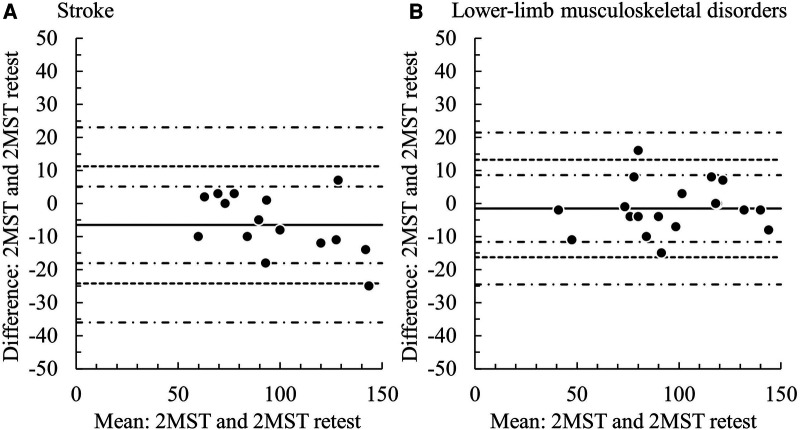
Bland–Altman plots of the test–retest 2MST. *Solid line*: the mean of the difference, *dotted line*: range of limit of agreement, *chain line*: 95% CI of the lower and upper limits of agreement. (**A**) stroke group, (**B**) lower–limb musculoskeletal disorders group.

Both groups demonstrated excellent results with ICC_1,1_ above 0.9 (stroke: 0.93, LMSD: 0.97). The SEM was 6.4 (95% CI: 4.7–10.1) in the stroke group and 5.3 (95% CI: −5.1–2.2) in the LMSD group (Table 5). From the Bland–Altman plots, a significant fixed error of approx. 6.5 steps increase on the retest was observed in the stroke group, although proportional errors were not significant, with the LoA ranging from −24.2 to 11.3 (Figure 3A and Table 5). The LoA for the stroke group in the 2MST was within an error width of ±19%, relative to the sample mean of 94.4 steps. No systematic error was observed in the LMSD group, and the LoA ranged from −16.2 to 13.3 (Figure 3B and Table 5). The LoA for the LMSD group in the 2MST was within an error width of ±15%, relative to the sample mean of 95.7 steps. The 95% CIs for the upper and lower bounds in the LoA for both groups were wide, and the estimates of the population parameters were not stable (Table 5).

**Table 5:** Results of relative and absolute reliability.

	Relative reliability		Absolute reliability				
	ICC_1,1_ (95% CI)	SEM (95% CI)	Fixed bias (95% CI) *p*-value	Proportional bias (*r*) *p*-value	LoA lower–upper	95% CI lower	95% CI upper
Stroke, *n* = 15	0.93 (0.81–0.98)	6.4 (4.7–10.1)	−6.5 (−11.5 to −1.5) 0.02	−0.49 0.06	−24.2–11.3	−36.0 to −18.0	5.1–23.1
LMSD, *n* = 19	0.97 (0.92–0.99)	5.3 (4.0–7.9)	−1.5 (−5.1–2.2) 0.41	0.11 0.66	−16.2–13.3	−24.5 to −11.6	8.6–21.5

ICC, intraclass correlation coefficient; SEM, standard error of measurement; 95% CI, 95% confidence interval; LoA, limits of agreement; LMSD, lower-limb musculoskeletal disorders.

## 4 Discussion

This study aimed to assess the validity and reliability of the 2MST as a tool for measuring the exercise endurance of individuals with stroke or an LMSD. The results indicated a moderate correlation between the 2MST and 6MWT in both groups, but the degree of congruence was insufficient. Although mobility and the 6MWT were correlated in both groups, no correlations with the 2MST were observed. The ICC for the 2MST was excellent in both groups, but only the stroke group exhibited a fixed bias of increased step count at retest. Based on these results, we assert that the 2MST is a valid and reliable tool for assessing the exercise endurance of individuals with stroke or LMSD. However, it is important to consider the potential for increased step count bias during retesting when assessing the exercise endurance in individuals with stroke.

The concurrent validity of the 2MST and the 6MST has already been confirmed in other diseases and populations (17–24), and our present findings extend the applicability of the 2MST as an assessment of exercise endurance. These results were obtained presumably because the participants were at least able to walk under supervision (FAC ≥ 3) and met the minimum unilateral lower-limb muscle strength required to perform the 2MST (capable of anti-gravity movements). Although the disease differs, it is known that some individuals with Parkinson's disease are unable to complete 2 min of marching (56), while those with mild walking disorders classified as Hoehn and Yahr stages I and II showed a correlation between the 2MST and 6MWT, and no correlation was observed in those with more severe walking disorders classified as stages III and IV (21).

These findings suggest that the present participants were appropriate for examining the concurrent validity of the 2MST and the 6MWT. However, our analyses revealed that the predicted ranges of the 6MWT estimated from the 2MST were wide, with 371.8 meters (±45%) for the stroke group and 285.7 (±58%) meters for the LMSD group, indicating insufficient congruence between the 2MST and 6MWT. This difference corresponds to the variations in the construct validity between the 2MST and 6MWT, which will be discussed later. In summary, although both the 2MST and 6MWT measure exercise endurance, they are performance tests that reflect different physical functions; therefore, the congruence between the 2MST and 6MWT is considered insufficient.

Interestingly, we observed that the 6MWT was associated with mobility and affected-limb muscle strength in the stroke group as well as mobility in the individuals with LMSD. The 2MST did not demonstrate a significant relationship. The 6MWT involves walking and is thus influenced by walking ability and other contributing factors, such as the muscle strength of the affected limb. In other words, it reflects not only exercise endurance but also walking ability and walking-related physical function. However, as the 2MST was not associated with mobility or muscle strength of the affected limb in this study, this result can be interpreted as an assessment focused on exercise endurance, independent of walking ability.

Previous research demonstrated that the 2MST is associated with the modified Rankin Scale, walking speed, and muscle strength in individuals with stroke (57). In individuals with knee osteoarthritis, pain intensity and physical function are associated with the 2MST (28). These findings are not in agreement with our present results, and there are several possible explanations for this discrepancy. In our study, the 2MST was administered in a stable environment with handrails, making it less likely that variations in mobility, physical function, and pain associated with stepping would affect the participants' test performance. We also observed that the pain intensity during walking was almost nonexistent in the individuals with stroke (NRS median, 0) and minimal in those with LMSD (NRS median, 1). An earlier investigation of individuals with knee osteoarthritis found pain to be more severe (NRS mean 8.12) (28). We thus propose that the 2MST performed with handrails is a method that easily cancels the influence of physical functions related to mobility and pain. Based on these considerations, we argue that the 6MWT and the 2MST should be selectively used depending on the situation and purpose. Given that the 6MWT is an established instrument with substantial evidence available, it should be prioritized 6MWT when possible. However, when environmental constraints or other factors make conducting the 6MWT challenging, the use of 2MST is justified. Furthermore, the 6MWT is appropriate for evaluating exercise endurance, including walking ability, whereas the 2MST is more suitable for evaluating exercise endurance with reduced influence from walking ability.

The relative reliability was excellent in both the present stroke and LMSD groups, comparable to or even better than that reported in previous studies (ICC: 0.83–0.945) that documented intra-rater reliability (14, 16, 20, 22, 26, 27). A notable point is that the range of the LoA in absolute reliability (±18% for stroke and ±15% for LMSD) was smaller than the values set alternatively by the 6MWT, i.e., ±35% for stroke (54) and ±18% for LMSD (55). Moreover, although there is limited evidence, recent investigations of the absolute reliability of the 2MST reported the range of LoA to be approx. ±32% for individuals with symptomatic peripheral artery disease (22) and approx. ±30% for individuals post-coronary revascularization (20). The LoA in our present study was superior for a performance test of exercise endurance. The high reliability of the assessment may be due to the well-trained physiotherapists, and the 2MST was conducted in a stable environment using handrails. It is also possible that not restricting the assessment by physiotherapists familiar with patients' conditions leads to high reliability. However, this approach may introduce examiner bias, and caution should be exercised in this regard. We also detected a fixed bias with an increase of 6.5 steps (∼8%) during the retest for the individuals with stroke, which could be interpreted as a learning effect. Since the result of 2MST was not blinded to the participants in this study, the learning effect is more likely to be induced in the retest. It is known that for older adults, the number of steps in the 2MST significantly increases in the third test compared with the first (16). Other studies of the absolute reliability of the 2MST described no systematic error in individuals with symptomatic peripheral artery disease (22). However, there was an increase of 7.5–7.7 steps (∼11%) on retest for individuals post-coronary revascularization (20). Similarly, learning effects upon retesting have been suggested in the 6MWT in individuals with stroke and hip fracture (54, 55). It remains unclear which participant characteristics are more likely to produce learning effects, but at least for individuals with stroke undergoing the 2MST, a careful interpretation of results considering fixed bias is warranted. According to the bias risk assessment tool for reliability and measurement error developed by COSMIN, note that not blinding both the examiner and the participants to the test results causes a risk of bias (58).

This study has several limitations. We did not examine the concurrent validity of exercise capacity by investigating its relationship with maximal or peak oxygen uptake. The %HRR in both tests was between 15% and 20%, indicating a low exercise load. In individuals with heart failure and morbid obesity, the concurrent validity between the peak oxygen uptake and the 2MST has been reported (18, 25). To examine the validity of the 2MST as a more rigorous assessment of exercise capacity based on exercise endurance, future studies including exhaled gas analyses are needed. Additionally, the assessments that we used for structural validity were mostly simple ones, and a replication study using more sensitive interval scales (such as walking speed or handheld dynamometry) is needed. Moreover, the absolute reliability remains a preliminary result due to the small sample size, and the 95% CI for the LoA was large. Although the sample size for Bland–Altman analyses remains a topic of debate (51), sample sizes of 100 or 200 are traditionally considered necessary to reflect population characteristics (46).

Lastly, a sensitivity analysis was not performed in this study. Constructing subgroups from a larger sample size and performing a sensitivity analysis are desired to examine the consistency of our results and provide more clinically interpretable and concrete findings. Despite these limitations, the strength of this study is providing externally valid results from a multicenter collaboration data. This is the first study to examine the validity and reliability of the 2MST as an assessment of exercise endurance in individuals with stroke or LMSD, offering evidence to promote the clinical application of this convenient test. Systematic reviews of the 2MST have indicated a lack of evidence of reliability, particularly absolute reliability (59), and our present study provides valuable foundational knowledge for future research.

## 5 Conclusions

Our research findings demonstrated that the 2MST is a valid and reliable method for assessing the exercise endurance of individuals with stroke or an LMSD. It is important to validate absolute reliability using a larger sample size, and when testing individuals with stroke, it may be necessary to consider the potential bias of increased step counts during retesting.

## Data Availability

The raw data supporting the conclusions of this article will be made available by the authors, without undue reservation.

## References

[1] Burtscher M. Exercise limitations by the oxygen delivery and utilization systems in aging and disease: coordinated adaptation and deadaptation of the lung-heart muscle axis—a mini-review. Gerontology. (2013) 59:289–96. 10.1159/000343990 23182831

[2] Kokkinos P Sheriff H Kheirbek R. Physical inactivity and mortality risk. Cardiol Res Pract. (2011) 2011:924945. 10.4061/2011/924945 21318105 PMC3034999

[3] Bull FC Al-Ansari SS Biddle S Borodulin K Buman MP Cardon G World health organization 2020 guidelines on physical activity and sedentary behaviour. Br J Sports Med. (2020) 54:1451–62. 10.1136/bjsports-2020-102955 33239350 PMC7719906

[4] Butland RJ Pang J Gross ER Woodcock AA Geddes DM. Two-, six-, and 12-minute walking tests in respiratory disease. Br Med J. (1982) 284:1607–8. 10.1136/bmj.284.6329.1607 6805625 PMC1498516

[5] Sullivan JE Crowner BE Kluding PM Nichols D Rose DK Yoshida R Outcome measures for individuals with stroke: process and recommendations from the American physical therapy association neurology section task force. Phys Ther. (2013) 93:1383–96. 10.2522/ptj.20120492 23704035

[6] McDonough CM Harris-Hayes M Kristensen MT Overgaard JA Herring TB Kenny AM Physical therapy management of older adults with hip fracture. J Orthop Sports Phys Ther. (2021) 51:CPG1–81. 10.2519/jospt.2021.0301 33522384

[7] Coleman G Dobson F Hinman RS Bennell K White DK. Measures of physical performance. Arthritis Care Res. (2020) 72(Suppl 10):452–85. 10.1002/acr.24373 33091270

[8] Cabinet Office, Government of Japan. Annual Report on Aging Society 2023. *Cabinet Office, Government of Japan*. Available online at: https://www8.cao.go.jp/kourei/whitepaper/w-2023/html/zenbun/s1_1_2.html (accessed December 1, 2023).

[9] Cabinet Office, Government of Japan. Annual Report on Aging Society 2022. *Cabinet Office, Government of Japan*. Available online at: https://www8.cao.go.jp/kourei/whitepaper/w-2022/html/zenbun/s1_2_2.html (accessed December 1, 2023).

[10] Yamada M Arai H. Long-term care system in Japan. Ann Geriatr Med Res. (2020) 24:174–80. 10.4235/agmr.20.0037 32829572 PMC7533196

[11] Population Division, United Nations. World population prospects 2022. *World Population Prospects* 2022. Available online at: https://population.un.org/wpp/ (accessed December 1, 2023).

[12] Seron P Oliveros M-J Gutierrez-Arias R Fuentes-Aspe R Torres-Castro RC Merino-Osorio C Effectiveness of telerehabilitation in physical therapy: a rapid overview. Phys Ther. (2021) 101:1–18. 10.1093/ptj/pzab053 PMC7928601 33561280

[13] Zheng J Hou M Liu L Wang X. Knowledge structure and emerging trends of telerehabilitation in recent 20 years: a bibliometric analysis via CiteSpace. Front Public Health. (2022) 10:904855. 10.3389/fpubh.2022.904855 35795695 PMC9251196

[14] Rikli RE Jessie Jones C. Development and validation of a functional fitness test for community-residing older adults. J Aging Phys Act. (1999) 7:129–61. 10.1123/japa.7.2.129

[15] Rikli RE Jessie Jones C. Functional fitness normative scores for community-residing older adults, ages 60–94. J Aging Phys Act. (1999) 7:162–81. 10.1123/japa.7.2.162

[16] Miotto JM Chodzko-Zajko WJ Reich JL Supler MM. Reliability and validity of the fullerton functional fitness test: an independent replication study. J Aging Phys Act. (1999) 7:339–53. 10.1123/japa.7.4.339

[17] Berlanga LA Matos-Duarte M Abdalla P Alves E Mota J Bohn L. Validity of the two-minute step test for healthy older adults. Geriatr Nurs. (2023) 51:415–21. 10.1016/j.gerinurse.2023.04.009 37146558

[18] Węgrzynowska-Teodorczyk K Mozdzanowska D Josiak K Siennicka A Nowakowska K Banasiak W Could the two-minute step test be an alternative to the six-minute walk test for patients with systolic heart failure? Eur J Prev Cardiol. (2016) 23:1307–13. 10.1177/2047487315625235 26743588

[19] Oliveros MJ Seron P Román C Gálvez M Navarro R Latin G Two-minute step test as a complement to six-minute walk test in subjects with treated coronary artery disease. Front Cardiovasc Med. (2022) 9:848589. 10.3389/fcvm.2022.848589 35615563 PMC9124827

[20] Chow JJL Fitzgerald C Rand S. The 2 min step test: a reliable and valid measure of functional capacity in older adults post coronary revascularisation. Physiother Res Int. (2023) 28:e1984. 10.1002/pri.1984 36428264

[21] Mollinedo-Cardalda I Cancela-Carral JM. The 2-minute step test: its applicability in the evaluation of balance in patients diagnosed with Parkinson’s disease. Top Geriatr Rehabil. (2022) 38:42–8. 10.1097/TGR.0000000000000341

[22] Braghieri HA Kanegusuku H Corso SD Cucato GG Monteiro F Wolosker N Validity and reliability of 2-min step test in patients with symptomatic peripheral artery disease. J Vasc Nurs. (2021) 39:33–8. 10.1016/j.jvn.2021.02.004 34120695

[23] Srithawong A Poncumhak P Manoy P Kumfu S Promsrisuk T Prasertsri P The optimal cutoff score of the 2-min step test and its association with physical fitness in type 2 diabetes mellitus. J Exerc Rehabil. (2022) 18:214–21. 10.12965/jer.2244232.116 35846235 PMC9271641

[24] Pedrosa R Holanda G. Correlation between the walk, 2-minute step and TUG tests among hypertensive older women. Braz J Phys Ther. (2009) 13:252–6. 10.1590/S1413-35552009005000030

[25] Ricci PA Cabiddu R Jürgensen SP André LD Oliveira CR Di Thommazo-Luporini L Validation of the two-minute step test in obese with comorbibities and morbidly obese patients. Braz J Med Biol Res. (2019) 52:e8402. 10.1590/1414-431X20198402 31482976 PMC6720022

[26] Nogueira MA Almeida TDN Andrade GS Ribeiro AS Rêgo AS Dias RdS Reliability and accuracy of 2-minute step test in active and sedentary lean adults. J Manipulative Physiol Ther. (2021) 44:120–7. 10.1016/j.jmpt.2020.07.013 33431278

[27] de Jesus SFC Bassi-Dibai D Pontes-Silva A da Silva de Araujo A de Freitas Faria Silva S Veneroso CE Construct validity and reliability of the 2-minute step test (2MST) in individuals with low back pain. BMC Musculoskelet Disord. (2022) 23:1062. 10.1186/s12891-022-06050-w 36471309 PMC9721032

[28] de Morais Almeida TF Dibai-Filho AV de Freitas Thomaz F Lima EAA Cabido CET. Construct validity and reliability of the 2-minute step test in patients with knee osteoarthritis. BMC Musculoskelet Disord. (2022) 23:159. 10.1186/s12891-022-05114-1 35177048 PMC8855541

[29] Mokkink LB Terwee CB Patrick DL Alonso J Stratford PW Knol DL The COSMIN study reached international consensus on taxonomy, terminology, and definitions of measurement properties for health-related patient-reported outcomes. J Clin Epidemiol. (2010) 63:737–45. 10.1016/j.jclinepi.2010.02.006 20494804

[30] Gagnier JJ Lai J Mokkink LB Terwee CB. COSMIN reporting guideline for studies on measurement properties of patient-reported outcome measures. Qual Life Res. (2021) 30:2197–218. 10.1007/s11136-021-02822-4 33818733

[31] Faul F Erdfelder E Buchner A Lang A-G. Statistical power analyses using G*power 3.1: tests for correlation and regression analyses. Behav Res Methods. (2009) 41:1149–60. 10.3758/BRM.41.4.1149 19897823

[32] Zou GY. Sample size formulas for estimating intraclass correlation coefficients with precision and assurance. Stat Med. (2012) 31:3972–81. 10.1002/sim.5466 22764084

[33] Charlson ME Pompei P Ales KL MacKenzie CR. A new method of classifying prognostic comorbidity in longitudinal studies: development and validation. J Chronic Dis. (1987) 40:373–83. 10.1016/0021-9681(87)90171-8 3558716

[34] Quan H Li B Couris CM Fushimi K Graham P Hider P Updating and validating the Charlson comorbidity index and score for risk adjustment in hospital discharge abstracts using data from 6 countries. Am J Epidemiol. (2011) 173:676–82. 10.1093/aje/kwq433 21330339

[35] Holden MK Gill KM Magliozzi MR Nathan J Piehl-Baker L. Clinical gait assessment in the neurologically impaired. Reliability and meaningfulness. Phys Ther. (1984) 64:35–40. 10.1093/ptj/64.1.35 6691052

[36] Collen FM Wade DT Robb GF Bradshaw CM. The rivermead mobility index: a further development of the rivermead motor assessment. Int Disabil Stud. (1991) 13:50–4. 10.3109/03790799109166684 1836787

[37] Maeshima S Yuzuki O Kobayashi T Koyama A Moriyasu M Osawa A. [Reliability and validity of the Japanese version of rivermead mobility index] rivermead mobility index nihongoban no sakusei to sono shiyou nituite (in Japanese). Sogo Rehabil. (2005) 33:875–9. 10.11477/mf.1552100180

[38] Williamson A Hoggart B. Pain: a review of three commonly used pain rating scales. J Clin Nurs. (2005) 14:798–804. 10.1111/j.1365-2702.2005.01121.x 16000093

[39] Kleyweg RP van der Meché FG Schmitz PI. Interobserver agreement in the assessment of muscle strength and functional abilities in Guillain-Barré syndrome. Muscle Nerve. (1991) 14:1103–9. 10.1002/mus.880141111 1745285

[40] Demeurisse G Demol O Robaye E. Motor evaluation in vascular hemiplegia. Eur Neurol. (1980) 19:382–9. 10.1159/000115178 7439211

[41] Collin C Wade D. Assessing motor impairment after stroke: a pilot reliability study. J Neurol Neurosurg Psychiatry. (1990) 53:576–9. 10.1136/jnnp.53.7.576 2391521 PMC488133

[42] ATS Committee on Proficiency Standards for Clinical Pulmonary Function Laboratories. ATS statement: guidelines for the six-minute walk test. Am J Respir Crit Care Med. (2002) 166:111–7. 10.1164/ajrccm.166.1.at1102 12091180

[43] Borg GA. Psychophysical bases of perceived exertion. Med Sci Sports Exerc. (1982) 14:377–81. 10.1249/00005768-198205000-00012 7154893

[44] Shrout PE Fleiss JL. Intraclass correlations: uses in assessing rater reliability. Psychol Bull. (1979) 86:420–8. 10.1037//0033-2909.86.2.420 18839484

[45] Stratford PW Goldsmith CH. Use of the standard error as a reliability index of interest: an applied example using elbow flexor strength data. Phys Ther. (1997) 77:745–50. 10.1093/ptj/77.7.745 9225846

[46] Bland M. Frequently asked questions on the design and analysis of measurement studies. *Martin Bland’s Home Page*. Available online at: https://www-users.york.ac.uk/∼mb55/meas/comfaq.htm (accessed January 15, 2024).

[47] Schober P Boer C Schwarte LA. Correlation coefficients: appropriate use and interpretation. Anesth Analg. (2018) 126:1763–8. 10.1213/ANE.0000000000002864 29481436

[48] Koo TK Li MY. A guideline of selecting and reporting intraclass correlation coefficients for reliability research. J Chiropr Med. (2016) 15:155–63. 10.1016/j.jcm.2016.02.012 27330520 PMC4913118

[49] Bland JM Altman DG. Statistical methods for assessing agreement between two methods of clinical measurement. Lancet. (1986) 1:307–10. 10.1016/S0140-6736(86)90837-8 2868172

[50] Abu-Arafeh A Jordan H Drummond G. Reporting of method comparison studies: a review of advice, an assessment of current practice, and specific suggestions for future reports. Br J Anaesth. (2016) 117:569–75. 10.1093/bja/aew320 27799171

[51] Gerke O. Reporting standards for a Bland–Atman agreement analysis: a review of methodological reviews. Diagnostics. (2020) 10:334. 10.3390/diagnostics10050334 32456091 PMC7278016

[52] Olofsen E. Webpage for Bland–Altman analysis. *Department of Anesthesiology of the LUMC* Available online at: https://sec.lumc.nl/method_agreement_analysis/ (accessed January 10, 2023).

[53] Olofsen E Dahan A Borsboom G Drummond G. Improvements in the application and reporting of advanced Bland–Altman methods of comparison. J Clin Monit Comput. (2015) 29:127–39. 10.1007/s10877-014-9577-3 24806333

[54] Liu J Drutz C Kumar R McVicar L Weinberger R Brooks D Use of the six-minute walk test poststroke: is there a practice effect? Arch Phys Med Rehabil. (2008) 89:1686–92. 10.1016/j.apmr.2008.02.026 18760152

[55] Overgaard JA Larsen CM Holtze S Ockholm K Kristensen MT. Interrater reliability of the 6-minute walk test in women with hip fracture. J Geriatr Phys Ther. (2017) 40:158–66. 10.1519/JPT.0000000000000088 27213999

[56] Cancela JM Ayán C Gutiérrez-Santiago A Prieto I Varela S. The senior fitness test as a functional measure in Parkinson’s disease: a pilot study. Parkinsonism Relat Disord. (2012) 18:170–3. 10.1016/j.parkreldis.2011.09.016 21968034

[57] Taylor-Piliae RE Latt LD Hepworth JT Coull BM. Predictors of gait velocity among community-dwelling stroke survivors. Gait Posture. (2012) 35:395–9. 10.1016/j.gaitpost.2011.10.358 22119886 PMC4696768

[58] Mokkink LB Boers M van der Vleuten CPM Bouter LM Alonso J Patrick DL COSMIN risk of bias tool to assess the quality of studies on reliability or measurement error of outcome measurement instruments: a delphi study. BMC Med Res Methodol. (2020) 20:293. 10.1186/s12874-020-01179-5 33267819 PMC7712525

[59] Bohannon RW Crouch RH. Two-minute step test of exercise capacity: systematic review of procedures, performance, and clinimetric properties. J Geriatr Phys Ther. (2019) 42:105–12. 10.1519/JPT.0000000000000164 29210933

